# Antiviral therapy effectively improves liver hemodynamics as evidenced by serum biomarker and contrast-enhanced ultrasound examinations in patients with hepatitis B cirrhosis

**DOI:** 10.7717/peerj.5484

**Published:** 2018-09-12

**Authors:** Xiaoyong Xu, Chaoxue Zhang, Chen Shi, Naizhong Hu, Bin Sun, Derun Kong, Jianming Xu

**Affiliations:** 1 Department of Gastroenterology, The First Affiliated Hospital of Anhui Medical University, Hefei, China; 2 Department of Ultrasound, The First Affiliated Hospital of Anhui Medical University, Hefei, China

**Keywords:** Hepatitis B virus, Cirrhosis

## Abstract

**Background and Aims:**

To prospectively evaluate the effects of antiviral therapy on liver hemodynamics in patients with hepatitis B cirrhosis.

**Methods:**

Seventy consecutive eligible HBV-related cirrhotic inpatients were enrolled in the prospective**** study. Fifty-two received different nucleoside analogs monotherapy and 18 denied antiviral therapy. Their liver biochemistry profiles and HBV-DNA were measured at the baseline and every 3 months. Peripheral blood vWF and sCD163, as well as liver ultrasound Doppler parameters including portal vein diameter (PVD), portal vein velocity (PVV), portal vein congestion index (PV-CI), hepatic vein damping index (HV-DI), hepatic arterial arrival time (HAAT), hepatic vein arrival time (HVAT) and intrahepatic cycle time (HV-HA), were measured at the baseline and the follow-up periods.

**Results:**

In**** the**** antiviral group, all patients achieved complete virologic and liver biochemical responses after 3-month antiviral treatment. Furthermore, the response states were maintained till the follow-up endpoint. However, in the non-antiviral group, HBV DNA replication resulted in higher levels of ALT and AST compared to the baseline values (*P* < 0.05). In the antiviral group, PVD, PV-CI, HV-DI, vWF-Ag and sCD163 were all significantly reduced than the baseline values (*P* < 0.05), and PVV was significantly increased than the baseline value (*P* < 0.05).

**Conclusions:**

Antiviral therapy could effectively suppress hepatocyte inflammation and alleviate the dysfunction of intrahepatic vascular endothelial and hepatic macrophages, which might improve hepatic hemodynamic function in HBV-related cirrhosis.

## Introduction

Hepatitis B virus (HBV)-associated cirrhosis with portal hypertension is a life-threatening condition. With the progression of portal hypertension, decompensated liver damage gradually exaggerates and the 5-year survival rate for patients is only 14% to 28% (Fattovich et al., 1995; Realdi et al., 1994). Hepatic venous pressure gradient (HVPG) is the traditional gold standard to assess portal hypertension. However, measurement of HVPG is invasive, expensive, and only available in a few specialized centers. Therefore, there have been extensive studies toward finding effective and noninvasive approaches to evaluate portal hypertension by focusing on a panel of biochemical markers and Doppler ultrasound methods.

Oral antiviral nucleoside analogs have been proved to improve the liver function of HBV-related cirrhotic patients. Lamivudine is an analogue of cytidine which can inhibit the reverse transcriptase of hepatitis B virus. Its long-term therapy could reduce the chance of decompensation for patients with chronic hepatitis B and decrease the incidence of liver cancer. It could also delay the clinical progression in patients with advanced liver fibrosis and cirrhosis (Liaw et al., 2004). However, few clinical studies have been reported about whether long-term antiviral therapy could influence liver hemodynamics, serological biomarkers, and portal hypertension in HBV-associated cirrhotic patients.

Increased vascular tone and the activation of hepatic macrophages caused by the disarrangement of intrahepatic microcirculation are important mechanisms in the development of portal hypertension in patients cirrhosis (Iwakiri & Groszmann, 2007; Matei et al., 2006; Holland-Fischer et al., 2011). Several recent large-scale studies had found that Von Willebrand factor (vWF), a peripheral intrahepatic vascular endothelial dysfunction marker, and soluble CD163(sCD163), a Kupffer cell activation marker, were both associated with portal hypertension, its staging, Child-Pugh score, and prognosis in patients with cirrhosis (La Mura et al., 2011; Ferlitsch et al., 2012; Grønbaek et al., 2012; Waidmann et al., 2013).

Doppler ultrasound can be a simple and non-invasive, as well as inexpensive and accurate, method to detect portal system hemodynamics. Contrast-enhanced ultrasound (CEUS) technique using microbubble ultrasound contrast agent could enhance the ultrasonic reflection and quantitatively evaluate hepatic hemodynamic perfusion. Some studies (Kim et al., 2007; Baik, 2010; Kim et al., 2012) found that several Doppler ultrasound parameters, such as hepatic vein damping index (HV-DI) and hepatic vein arrival time (HVAT), were associated with HVPG and were able to reveal the alterations of portal vein pressure in cirrhotic patients.

Therefore, the objective of current prospective study was to evaluate the effects of the long-term antiviral therapy on vWF**,** sCD163, and liver hemodynamics. We demonstrated that long-term antiviral therapy could improve hepatic hemodynamic dysfunction by decreasing hepatic macrophages activation and alleviating intrahepatic vascular endothelial dysfunction in cirrhotic patients with active HBV replication and liver inflammation.

## Methods

### Ethics Statement

The prospective cohort study was approved by the Ethics Committee at the First Affiliated Hospital of Anhui Medical University (Ref #: 20110910). The study protocol was explained to every patient, and the written informed consent was obtained before the beginning of the study.

### Study population

From July 2012 to February 2015, inpatients with HBV-related cirrhosis from the Department of Gastroenterology at the First Affiliated Hospital of Anhui Medical University were consecutively enrolled. Inclusion criteria for this study included: 1, diagnosis of liver cirrhosis based on clinical, histology, ultrasonography, computed tomography (CT) or magnetic resonance imaging (MRI); 2, positive HBsAg for ≥12 months; 3, age between18 to 75 years old; 4, elevated serum aspartate (AST) and/or alanine aminotransaminase (ALT) levels on ≥ 2 times during the previous 12 months; 5, serum HBV DNA (>1,000 copies/mL) detectable by polymerase chain reaction (PCR) (Fluorescence Quantitation kit; Shanghai ZJ Bio-Tech Co., Ltd., Shanghai, China). Exclusion criteria included: 1, patients with active alcohol consumption, co-infections, or chronic liver disease from other causes; 2, history of current therapy with antivirals; 3, baseline level of creatinine >1.5 times above the upper limit of normal; 4, previous operation for portal hypertension or transjugular intrahepatic porto-systemic stent shunt placement; 5, hepatocellular carcinoma or other neoplastic diseases, severe cardiopulmonary disease, spontaneous bacterial peritonitis, obvious portal or splenic vein thrombosis; 6, previous endoscopic sclerosis or band ligation of EV within one month before the current study; 7, under treatment with vasoactive drugs such as β-blocker, somatostatin and its analogues, or diuretics within one week before the Doppler ultrasound examination; 8, acute bleeding from esophageal varices; 9, current pregnancy or lactation; 10, patients whose hepatic veins could not be identified convincingly on US. Hepatic decompensation was defined when patients had theChild-Pugh scores ≥ 8 or had ≥ 1 of the following events: ascites, hepatic encephalopathy, portal hypertensive bleeding.

### Study design

All eligible patients underwent Doppler ultrasound and CEUS examinations. Peripheral vWF-Ag and sCD163 were detected by the ELISA method before initiation of antiviral therapy. These eligible patients were assigned into antiviral therapy group or non-antiviral therapy group. Antiviral therapy group received antiviral treatments after study enrollment: lamivudine (LAM) (100 mg/d; Suzhou Pharmaceutical Co., Ltd., Suzhou, China; GlaxoSmithKline, Brentford, UK), adefovir (ADV) (10 mg/d; Tianjin Pharmaceutical Co., Ltd., Tianjin, China; GlaxoSmithKline, Brentford, UK), entecavir (ETV) (0.5 mg/d, Bristol-Myers Squibb, Redwood City, CA, USA). Non-antiviral therapy group refused the above antiviral treatments for reasons including high costs of long-term treatment, resistance and adverse effects. They only received symptomatic treatment (analgesics for pain, antipyretics for fever, antitussives for cough).

**Figure 1:**
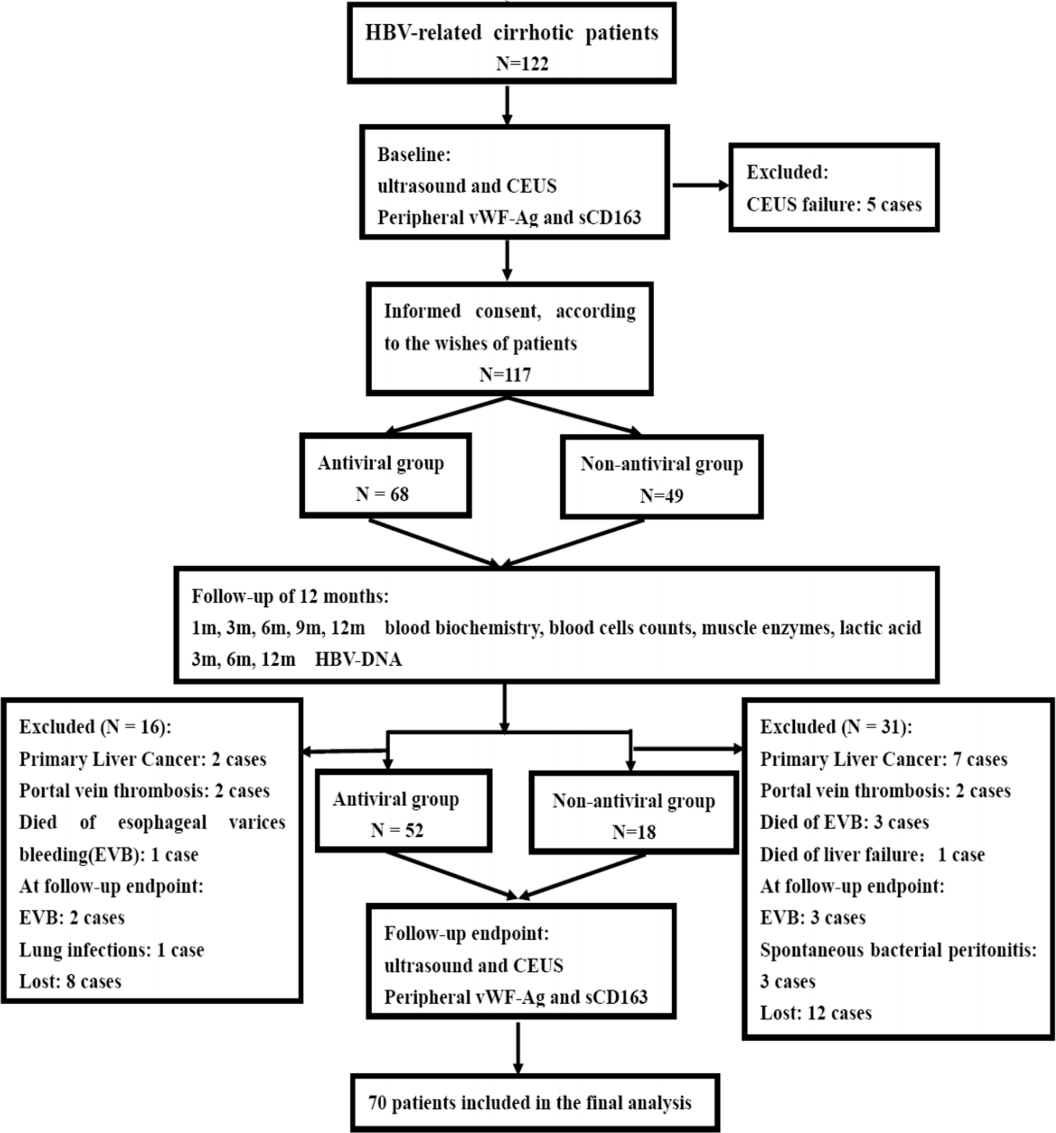
** Diagram illustrating study design and procedure.**

Patients were followed up for the clinical evaluations and routine laboratory tests at 1, 3, 6, 9 and 12 months: serum HBV DNA was measured at 3, 6 and 12 months; Doppler ultrasound and CEUS examinations; peripheral vWF-Ag and sCD163 levels were tested at beginning (baseline) and 12 months (follow-up endpoint). An upper esophageal gastrointestinal endoscopy was performed in all patients to assess the presence of esophageal varices. All patients received standard treatments with beta-blockers, diuretics, polyene phosphatidylcholine, glutathione, diammonium glycyrrhizinate and endoscopic therapy, when indicated. Endoscopic band ligation/sclerotherapy and beta-blockers were used for those patients, if necessay. Endoscopic procedures were repeated every 2 weeks until the esophageal varices resolved or were too small for further treatment. Endoscopic follow-up was performed at 3-month intervals during the first year. Beta-blockers and diuretics treatments werediscontinued 1-week before Doppler ultrasound and serological markers measurements. During the follow-up study, those cases would be excluded from the study if these patients had the following conditions, including hepatocellular carcinoma, portal or splenic vein thrombosis, death, serious adverse events of antiviral drugs, gastrointestinal bleeding or infection at follow-up endpoint, unable to complete the ultrasound examination, and lost (Fig. 1).

### Doppler ultrasound and contrast-enhanced ultrasound examinations

The upper abdominal Doppler ultrasonography examination was performed by one operator with more than 15 years’ experience (C.X.Zhang) in ultrasonography. After fasting for more than 6 hours, patients were examined for portal vein diameter (PVD), mean portal vein velocity (PVV), HV-DI. Portal vein congestion index (PV-CI) was calculated by *π* ×portal vein radius^2^/mean portal vein velocity (Moriyasu et al., 1986). HV Doppler waveforms were documented from three repeated measurements. Maximum and minimum velocities of downward HV flow at the right HV were recorded at the longitudinal scanning planes. HV-DI was calculated by the formula of minimum velocity/ maximum velocity of downward HV flow (Kim et al., 2007).

CEUS was performed by hospital experienced sonographer with a LOGIQ 7 system (GE Healthcare, WI, USA) in the coded phase inversion mode with a 4C convex array transducer (frequency of 3 to 6 MHz, mechanical index of 0.08 and scanning depth of 12 to 14 centimeters). Portal vein was identified and labelled by a single focus point. SonoVue (Bracco SpA, Milan, Italy), a second-generation contrast agent which contained sulfur hexafluoride–filled microbubbles, was used.

Patients received the examinations at the supine position. After the traditional B-mode and color Doppler sonographic examinations, the sonogram probe was placed along the anterior axillary andmidaxillary lines. Portal vein, hepatic artery, and right or middle hepatic vein were scanned simultaneously in a cross plane. Then, a real-time dual-frame CEUS examination was performed at a low mechanical index of 0.08. Baseline HV signals were first documented for 10 seconds, and then a 2.4 mL SonoVue bolus was injected for 1 second and followed immediately by a rapid 5 ml normal saline flush through a three-way catheter for 2 seconds and a 20G intravenous needle inserted into the cubital vein at the left antecubital fossa. Time to inject SonoVue was recorded by the equipped computer software. Data were acquired till 60 seconds after the contrast injection. Patients were instructed to hold their breathes in the end-expiration phase for 20 seconds from 5 seconds after the contrast injection. Time delay from the contrast injection till the first echogenic bubbles of the contrast agent observed in the hepatic artery and hepatic vein were measured. This was defined as the arrival times for the contrast agent (hepatic artery arrival time HAAT and hepatic vein arrival time HVAT). To reduce the influence from the blood circulation cycles, we applied the hepatic vein–artery time interval (hepatic vein arrival time minus hepatic artery arrival time HV-HA) as the intrahepatic circulatory time. All of the videotapes from each measurement were re-analyzed by another operator in order to assess the interobserver variability (Zhang et al., 2011). The results showed that two observers had statistically significant correlations during the contrast agent arrival time measurements in the hepatic artery (*r* = 0.921, *P* < 0.001) and hepatic vein (*r* = 0.918, *P* < 0.001). All of the CEUS procedures were performed by the same examiner (C.X.Zhang).

### Peripheral vWF-Ag and sCD163 determination

Blood samples were drawn from peripheral vein into a heparin tube 2 days before the ultrasound examination. After centrifugation at 3,000 g at 4 °C for 20 min, plasma was collected and placed at −80 °C for further analysis. vWF-Ag and sCD163 were measured by an ELISA system using vWF-ELISA assay kit (Lot E-11793; Nanjing Jiancheng Technology Co., Ltd., Nanjing, China) and sCD163-ELISA assay kit (Lot CK-E11504H; Nanjing Jiancheng Technology Co., Ltd., Nanjing, China) based on the instructions. Fifteen healthy volunteers with matched for sex and age and without history of chronic liver disease, cardiovascular and cerebrovascular diseases and recent medication were recruited as controls for the determinations of peripheral vWF-Ag and sCD163.

### Statistics

Statistical analyses were performed using SPSS 16.0 (SPSS, IBM, Armonk, NY, USA). Continuous values of normal distribution were presented as mean ± SD and comparisons between two groups were performed by two independent sample *t* test. Continuous values of non-normal distribution were expressed by median and comparisons between two groups were performed by Mann–Whitney non-parametric test. Categorical values were presented as count or proportions and compared by the *χ*2 test. All *P*-values reported were two-sided and a *P*-value <0.05 was considered statistically significant.

## Results

### Patient characteristics and study enrollment

From July 2012 to February 2015, 122 consecutive patients with proven HBV-related cirrhotic were screened and 5 were excluded for CEUS failure at baseline. One hundred seventeen cirrhotic patients were enrolled in the follow-up study. Sixty-eight patients who accepted were included in the antiviral therapy group, and 49 patients who refused antiviral therapy were included in the control group. During the follow-up study period, 16 patients in the antiviral therapy group and 31 patients in the non-antiviral therapy group were excluded. Ultimately, 70 patients who met the study criteria and completed the follow-up study were included in the final statistical analysis (see Fig. 1). Of these, 52 patients were administrated appropriate antiviral therapy, including ETV (*N* = 21), ADV (*N* = 3) and LAM (*N* = 28). Eighteen patients rejecting antiviral therapy were followed up as the control group. They received the same treatments as the antiviral group except antiviral therapy. The antiviral group and the control group were comparable in terms of their gender, age, liver functions, grade of EV, HBV DNA level, and liver Doppler ultrasound parameters, etc. (see Table 1).

**Table 1:** Demography of the patients (mean ± SD).

	Antiviral (*N* = 52)	Non-antiviral (*N* = 18)	*P* value
Gender (male/female)	43/9	13/5	0.338
Age (years)	48.10 ± 10.02	49.33 ± 10.42	0.656
Child-Pugh class (A/B/C)	26∕18∕8	9∕8∕1	0.509
EV(With/ without)	43/9	13/5	0.338
History of variceal bleeding (With/ without)	25/27	9/9	0.584
TB (mmol/L)	28.48 ± 23.96	28.13 ± 22.08	0.956
ALB (g/L)	33.32 ± 7.44	31.79 ± 7.04	0.449
ALT(IU/L)	113.00 ± 70.10	87.00 ± 46.66	0.148
AST(IU/L)	102.94 ± 61.80	73.72 ± 32.81	0.061
PT(s)	17.61 ± 3.21	16.83 ± 1.96	0.341
PLT (10^9^/L)	65.94 ± 40.13	71.11 ± 55.27	0.672
HBV-DNA median (copies/ml)	161,000 (1,230–84,800,000)	52,800 (1,770–5,930,000)	0.311
PVV (cm/s)	16.73 ± 4.56	16.85 ± 5.81	0.927
PVD (cm)	1.46 ± 0.22	1.45 ± 0.33	0.860
PV-CI (cm s)	0.11 ± 0.04	0.11 ± 0.04	0.749
HV-DI	0.68 ± 0.18	0.72 ± 0.19	0.421
HAAT (s)	11.12 ± 2.93	10.78 ± 2.67	0.669
HVAT (s)	18.40 ± 4.32	18.89 ± 3.46	0.668
HV-HA (s)	7.35 ± 2.54	8.11 ± 1.60	0.237

**Notes.**

EV Esophageal varices HBV DNA Hepatitis B virus DNA ALT Alanine aminotransferase AST Aspartate aminotransferase TB Total bilirubin ALB Serum albumin PT Prothrombin time PLT Platelet count

### Impact of antiviral treatment on serum biomarkers

**Figure 2:**
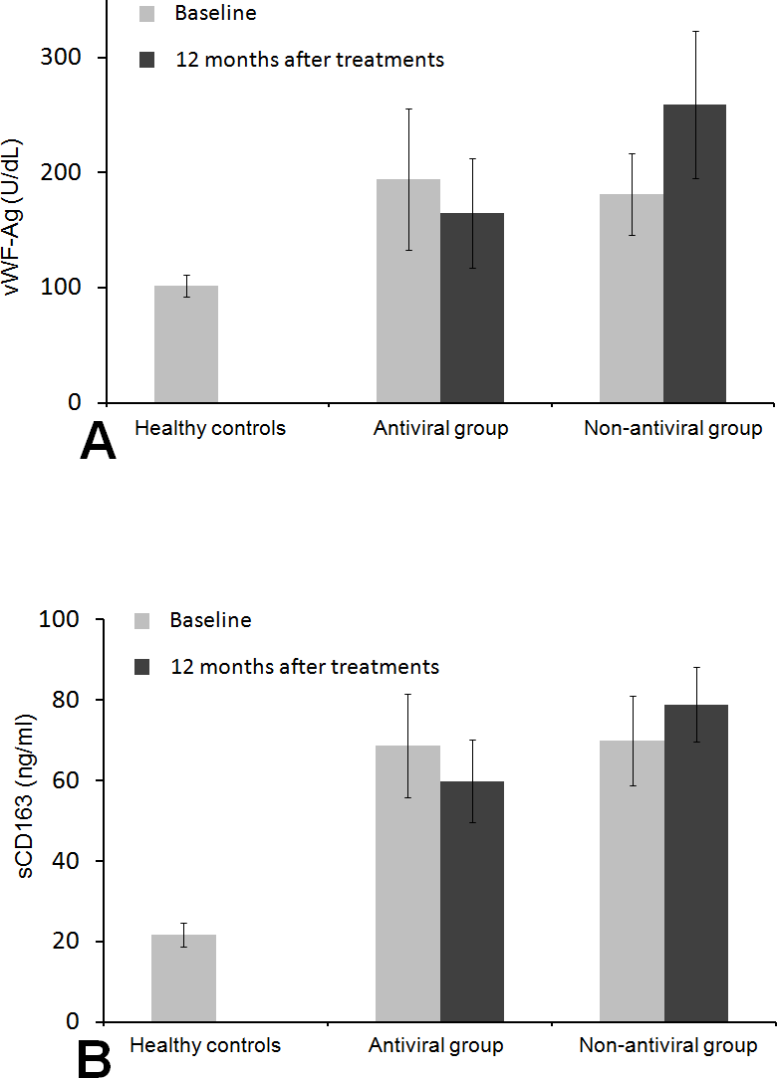
Histogram of levels of peripheral vWF-Ag and sCD16 in patients and healthy control subjects. Comparisons of vWF-Ag (A) and sCD163 (B) among different groups at baseline and at 12 months after the treatment. vWF-Ag and sCD163 were significantly higher in patients with antiviral therapy and non-antiviral therapy than those in healthy controls. The histograms represent mean value. *P < 0.05 compared to Non-antiviral group and Antiviral group. **P > 0.05 Non-antiviral group compared to Antiviral group.

In patients with antiviral therapy, baseline peripheral levels of vWF-Ag and sCD163 both significantly increased compared to the healthy controls (vWF-Ag 194.61 ± 61.38 U/dl, sCD163 68.71 ± 12.80 vs vWF-Ag 101.75 ± 9.28 U/dl, sCD163 21.72 ± 2.92 ng/ml in healthy controls, both *P* < 0.001). In patients with non-antiviral therapy, baseline peripheral levels of both vWF-Ag and sCD163 were also significantly higher compared to the healthy controls(vWF-Ag 181.41 ± 35.34 U/dl, sCD163 69.96  ± 11.15 ng/ml vs vWF-Ag 101.75 ± 9.28 U/dl, sCD163 21.72  ± 2.92 ng/ml in healthy controls, both *P* < 0.001). Baseline peripheral levels of vWF-Ag and sCD163 were not significantly different between the antiviral group and non-antiviral group (*P* = 0.273 and *P* = 0.714, respectively) (Fig. 2). After 12-month treatment, patients in the antiviral group had significant decreases in the vWF-Ag and sCD163 levels, whereas patients in the non-antiviral group had increased levels of vWF-Ag and sCD163 (Fig. 2).

### Impact of antiviral treatment on virological response and hepatic function

After antiviral treatment for 3 months, HBV DNA reduced to undetectable levels (defined as <1,000 copies/mL), and liver biochemical indicators, particularly ALT and AST, also gradually returned to the normal level in all patients. In addition, the virological response and liver biochemical alterations in patients receiving antiviral treatment were maintained until the end of the follow-up period. None of the control group spontaneously achieved undetectable HBV DNA, and the high viral replications in all non-antiviral patients were always detected at high levels during the study. Analysis of liver biochemical profiles (ALT, AST, ALB, TB and PT) showed steady improvements in the liver function in the antiviral group. However, non-antiviral group had significantly higher ALT, AST and PT (Table 2).

**Table 2:** Changes in biochemical measures in control patients receiving no treatment and in patients treated with antiviral treatment for 12 months (mean ± SD).

	Antiviral (*n* = 52)		*P* value	Non-antiviral (*n* = 18)		*P* value
	baseline	12 months		baseline	12 months	
TB (umol/L) (5.10–19.00)^a^	28.48 ± 23.96	15.31 ± 3.28	0.000	28.13 ± 22.08	30.73 ± 12.73	0.668
ALB (g/L) (40.0–55.0)^a^	33.32 ± 7.44	36.78 ± 4.08	0.021	31.79 ± 7.04	28.21 ± 3.48	0.097
ALT (IU/L) (9–50)^a^	113.00 ± 70.10	29.17 ± 9.80	0.000	87.00 ± 46.66	122.50 ± 33.81	0.013
AST (IU/L) (15–40)^a^	102.94 ± 61.80	30.73 ± 8.52	0.000	73.72 ± 32.81	105.11 ± 36.09	0.010
PT (s) (11.0–16.0)^a^	17.61 ± 3.21	15.88 ± 0.88	0.001	16.83 ± 1.96	18.52 ± 1.15	0.003
PLT(10^9^/L) (125–350)^a^	65.94 ± 40.13	70.46 ± 36.32	0.548	71.11 ± 55.27	48.22 ± 14.57	0.118

**Notes.**

a normal range.

### Impact of antiviral treatment on liver Doppler ultrasound parameters

As shown in Table 3, in antiviral treatment group, PVD, PV-CI and HV-DI decreased significantly compared to the baseline (*P* < 0.05), while the PVV was significantly greater than the baseline value (*P* < 0.05) after a sustained antiviral therapy for 12 months. However, there was a little bit increase of mean in each CEUS parameter of HAAT, HVAT and HV-HA in antiviral group compared with baseline, although without statistical significance (*P* > 0.05). Meanwhile, PV-CI, HV-DI, HVAT and HV-HA all significantly decreased compared to the baseline in the control group without antiviral treatment at 12 months (*P* < 0.05), whereas there were no significant differences in PVD, PVV and HAAT compared with baseline (*P* > 0.05).

**Table 3:** Changes in liver ultrasound parameters in patients with 12- month antiviral treatment and in patients without antiviral treatment (mean ± SD).

	Antiviral (*N* = 52)		*P* value	Non-antiviral (*N* = 18)		*P* value
	baseline	12 months		baseline	12 months	
PVV (cm/s)	16.73 ± 4.56	19.53 ± 2.34	0.000	16.85 ± 5.81	13.53 ± 2.74	0.074
PVD (cm)	1.46 ± 0.22	1.38 ± 0.14	0.025	1.45 ± 0.33	1.55 ± 0.26	0.327
PV-CI (cm s)	0.11 ± 0.04	0.08 ± 0.02	0.000	0.11 ± 0.04	0.15 ± 0.05	0.011
HV-DI	0.68 ± 0.18	0.57 ± 0.13	0.001	0.72 ± 0.19	0.87 ± 0.11	0.008
HAAT (s)	11.12 ± 2.93	11.65 ± 2.62	0.326	10.78 ± 2.67	9.89 ± 1.84	0.253
HVAT (s)	18.40 ± 4.32	19.75 ± 3.92	0.099	18.89 ± 3.46	15.28 ± 1.60	0.000
HV-HA (s)	7.35 ± 2.54	8.10 ± 2.30	0.118	8.11 ± 1.60	5.39 ± 1.14	0.000

### Impact of antiviral treatment on liver ultrasound parameters and peripheral serological biomarkers at different clinical stages of cirrhosis

As shown in Table 4, for both compensated cirrhosis patients and decompensated cirrhosis patients, antiviral therapy resulted in the improvements in the liver ultrasound hemodynamic parameters and peripheral serological biomarkers. Particularly, among decompensated cirrhosis, there were statistically significant differences in peripheral levels of vWF-Ag and sCD163 and in each of the ultrasound parameters except HAAT between at baseline and follow-up endpoint (all *P* < 0.05). However, in compensated cirrhosis, there were statistically significant reductions only in PV-CI and peripheral levels of vWF-Ag and sCD163 compared with baseline (all *P* < 0.05), whereas no significant differences in other ultrasound parameters compared with baseline (all *P* > 0.05).

**Table 4:** Changes in liver ultrasound parameters and peripheral serological biomarkers in patients at different clinical stages of cirrhotic after 12-month antiviral treatment (mean ± SD).

	Compensated cirrhosis (*N* = 13)		*P* value	Decompensated cirrhosis (*N* = 39)		*P* value
	baseline	12 months		baseline	12 months	
PVV (cm/s)	18.00 ± 4.28	20.08 ± 2.33	0.137	16.30 ± 4.63	19.35 ± 2.35	0.001
PVD (cm)	1.44 ± 0.26	1.36 ± 0.16	0.344	1.46 ± 0.21	1.38 ± 0.13	0.038
PV-CI (cm s)	0.10 ± 0.03	0.07 ± 0.02	0.031	0.11 ± 0.05	0.08 ± 0.02	0.000
HV-DI	0.55 ± 0.15	0.47 ± 0.12	0.134	0.72 ± 0.16	0.61 ± 0.12	0.001
HAAT (s)	12.85 ± 3.91	13.23 ± 3.19	0.786	10.54 ± 2.32	11.13 ± 2.20	0.253
HVAT (s)	22.46 ± 5.88	23.08 ± 5.28	0.781	17.05 ± 2.56	18.64 ± 2.60	0.008
HV-HA (s)	9.62 ± 3.01	9.85 ± 2.73	0.840	6.59 ± 1.86	7.51 ± 1.83	0.030
vWF-Ag (U/dl)	155.11 ± 27.32	132.48 ± 22.58	0.030	207.78 ± 64.10	175.81 ± 48.63	0.015
sCD163 (ng/ml)	59.00 ± 10.64	50.77 ± 7.47	0.032	71.95 ± 11.88	62.91 ± 9.24	0.000

### Esophageal varices progress

After antiviral therapy for 12 months, only two cases of the nine patients without EVs in the antiviral group had mild EVs. However, in the non-antiviral group, the extents of EVs in five patients became worse (two cases progressed to mild EVs., Three cases progressed to moderate-severe EVs including one patient experienced esophageal varices bleeding) (22.2% vs. 100.0%, *P* = 0.021).

## Discussion

To our knowledge, this prospective, cohort clinical investigation was the first study to assess whether long-term oral HBV nucleoside analogues antiviral therapy could improve liver hemodynamics of HBV-associated cirrhotic patients by Doppler ultrasound method.

Long-term antiviral therapy could delay the clinical progression of liver cirrhosis (Dienstag et al., 2003). Since fibrosis often irregularly distributes in cirrhotic liver, it is difficult to evaluate the degree of fibrosis accurately and consistently by liver pathology examination. Some studies supported the opinion that repeated HVPG measurements could be a better way to assess the response to antiviral therapy in patients with chronic hepatitis C and HCV-related cirrhosis (Rincon et al., 2006; Roberts et al., 2007). A prospective study from Greece reported that in HBV-associated cirrhosis with virus replication, monotherapy with lamivudine for 12 months reduced HVPG, with evidences of virological suppression and biochemical remission (Manolakopoulos et al., 2009). However, because of its invasiveness, high examination costs, technical equipment requirements, and difficulties in the follow-up, HVPG is not suitable for widespread routine clinical application to evaluate portal hypertension .

Doppler ultrasound with CEUS technology and peripheral serum biomarkers have the advantages of being non-invasive, simple and inexpensive to assess the cirrhotic haemodynamic dysfunctions and to evaluate the responses to medical managements of portal hypertension.

In the current study, HBV DNA decreased to undetectable levels in all patients of antiviral treatment (complete virologic response) after 3-month sustained antiviral treatment. Furthermore, the states of complete virologic response in all patients receiving antiviral treatment were maintained until the follow-up endpoint. Nobody in the control group achieved virologic response during the follow-up period. At the same time, together with the virologic response, the levels of ALT and AST in antiviral group also quickly returned to normal with complete biochemical response at 3 months, and these states in all patients were maintained until the follow-up endpoint. However, ALT, AST and PT remained higher in the non-antiviral treatment group, which suggested that the persistent viral replication could cause continuous inflammation of the liver cells. In addition, some studies had shown that the levels of peripheral vWF-Ag and sCD163 were positively correlated with portal vein pressure (La Mura et al., 2011; Grønbaek et al., 2012). We also found that the levels of vWF-Ag and sCD163 in cirrhotic patients were significantly increased than those of the healthy control group. In the non-antiviral group, peripheral levels of vWF-Ag and sCD163 at the follow-up endpoint were both significantly higher than the baseline levels. However, sustained antiviral treatment effectively reduced both levels regardless of cirrhosis stage. These evidences showed that the intrahepatic vascular endothelial dysfunction and activation of hepatic macrophages were associated with hepatocytes inflammatory activity caused by HBV replication. Sustained virologic response may alleviate the dysfunction of intrahepatic vascular endothelial and hepatic macrophages in HBV-related cirrhosis.

To evaluate the impact of antiviral therapy on the hepatic hemodynamics in HBV-related cirrhosis, we chose three main indicators: PV-CI, HV-DI and intrahepatic circulatory time (HV-HA), which represented the hemodynamics of portal vein, hepatic vein, and intrahepatic circulation, respectively. Several studies showed that PV-CI was positively correlated with HVPG, and the sensitivity and specificity for portal hypertension for a PV-CI cut-off value of 0.1 cm s were both 95% (Moriyasu et al., 1986; Haag et al., 1999). The Doppler HV waveform in the healthy subjects is triphasic (two negative and one positive waves), which is the consequence of variations in the central venous pressure due to the cardiac cycles. In cirrhotic patients, the abnormal biphasic or monophasic HV waveforms could appear. HV-DI could quantitate the extent of hepatic vein waveform abnormalities (loss of pulsatility) and assess the severity of portal hypertension. Kim et al. (Kim et al., 2007) found that HV-DI was significantly correlated to the grade of HVPG, and HV-DI >0.6 indicated significantly more severe portal hypertension in cirrhosis. Our previous study also found that shorter HV-HA was significantly correlated with higher free portal pressure in HBV-related cirrhosis (Zhang et al., 2011). In the current study, we found that the portal hemodynamic parameters and HV-DI had significant improvements in the antiviral therapy group, suggesting the alleviation of portal hypertension. However, no CEUS parameters in the antiviral group got better compared to baseline. By stratified analysis, we found that antiviral therapy had little effect on improving all ultrasound hemodynamic parameters in compensated cirrhosis except PV-CI. Whereas in decompensated cirrhosis, sustained antiviral therapy not only improved the portal hemodynamics parameters and HV-DI, but also improved HVAT and HV-HA. This showed that antiviral therapy could effectively improve severe hemodynamic disorders in portal hypertension, particularly in decompensated HBV-related cirrhosis. In contrast, the PV-CI, HV-DI, HVAT and HV-HA all significantly became worse in the control group, which indicated the worsening of portal vein hemodynamic abnormalities, more flat hepatic vein waveform, and shorter intrahepatic cycle times. This also suggested the aggravation of portal hypertension was due to the sustained high viral load and liver biochemical abnormalities. Our study found that antiviral therapy effectively postponed the emergence and development of esophageal varices. This was consistent with the results of a recent study (Li et al., 2013). Clearly, our study that antiviral therapy improved liver hemodynamics and portal hypertension associated serological biomarkers supported the conclusion.

One of the limitations of our study was that we did not compare our results with the change of HVPG for better understanding the evolution of portal hypertension during the antiviral therapy, because the repeated invasive and expensive measurements of HVPG during the follow-up periods were really difficult to be accepted by our patients. However, compared to the previous studies, some patients refused long-term antiviral therapy due to their subjective reasons, which allowed us to have a control group. Meanwhile, we included vWF-Ag and sCD163, which were both closely related to formation of portal hypertension, into our study. This helped us to better understand the impact of antiviral therapy on hemodynamic changes of cirrhosis and portal hypertension. Our results clearly confirmed that virologic response induced by long-term oral HBV nucleoside analogues monotherapy could effectively suppress hepatocyte inflammation and further alleviate the dysfunction of intrahepatic vascular endothelial and hepatic macrophages, might also improve liver hemodynamic dysfunction in HBV-related cirrhosis.

Another limitation was patient selection bias in that quite a number of patients in two groups, especially in the non-antiviral group, were excluded during the follow-up period. However, there were significant differences in any baseline parameters in patients included the final analysis and the patients excluded, which could reduce the influence of lost to follow-up bias on the result analysis.

Of the 52 antiviral therapy patients, 21 cases of patients accepted entecavir, and the remaining 31 cases accepted lamivudine or adefovir mainly due to the cost. All patients acquired complete virological response at week 12 and held until the follow-up endpoint without viral load rebound and serious adverse events. It seemed that there were no significant differences of antiviral efficacy and safety among lamivudine, adefovir and entecavir. However, we could not further observe the potential rebound of viral load caused by antiviral resistance in the antiviral treatment group, because our follow-up period was only 12 months and the sample size was relatively small. Therefore, further studies with a large sample size and long follow-up period are required to confirm our study results.

## Conclusions

In conclusion, antiviral therapy could effectively suppress hepatocyte inflammation and alleviate the dysfunction of intrahepatic vascular endothelial and hepatic macrophages, which might improve hepatic hemodynamic function in HBV-related cirrhosis.

##  Supplemental Information

10.7717/peerj.5484/supp-1 Data S1 Raw data

## Additional Information and Declarations

### Data Availability

The following information was supplied regarding data availability: The raw data are provided in Data S1.
